# Automatic Hemorrhage Detection From Color Doppler Ultrasound Using a Generative Adversarial Network (GAN)-Based Anomaly Detection Method

**DOI:** 10.1109/JTEHM.2022.3199987

**Published:** 2022-08-19

**Authors:** Jhimli Mitra, Jianwei Qiu, Michael MacDonald, Prem Venugopal, Kirk Wallace, Hossam Abdou, Michael Richmond, Noha Elansary, Joseph Edwards, Neerav Patel, Jonathan Morrison, Luca Marinelli

**Affiliations:** General Electric Research Niskayuna NY 12309 USA; Cognex Corporation Natick MA 01760 USA; School of MedicineUniversity of Maryland, Baltimore Baltimore MD 21201 USA

**Keywords:** Hemorrhage detection, color Doppler ultrasound, unsupervised anomaly detection, generative adversarial network, deep learning

## Abstract

Hemorrhage control has been identified as a priority focus area both for civilian and military populations in the United States because exsanguination is the most common cause of preventable death in hemorrhagic injury. Non-compressible torso hemorrhage (NCTH) has high mortality rate and there are currently no broadly available therapies for NCTH outside of a surgical room environment. Novel therapies, which include High Intensity Focused Ultrasound (HIFU) have emerged as promising methods for hemorrhage control as they can non-invasively cauterize bleeding tissue deep within the body without injuring uninvolved regions. A major challenge in the application of HIFU with color Doppler US guidance is the interpretation and optimization of the blood flow images in real-time to identify the hemorrhagic focus. Today, this task requires an expert sonographer, limiting the utility of this therapy in non-clinical environments. In this work, we investigated the feasibility of an automated hemorrhage detection method using a Generative Adversarial Network (GAN) for anomaly detection that learns a manifold of normal blood flow variability and subsequently identifies anomalous flow patterns that fall outside the learned manifold. As an initial feasibility study, we collected ultrasound color Doppler images of femoral arteries in an animal model of vascular injury (N = 11 pigs). Velocity information of the blood flow were extracted from the color Doppler images that were used for training and testing the anomaly detection network. Normotensive images from 8 pigs were used for training, and testing was performed on normotensive, immediately after injury, 10 minutes post-injury and 30 minutes post-injury images from 3 other pigs. The residual images or the reconstructed error maps show promise in detecting hemorrhages with an AUC of 0.90, 0.87, 0.62 immediately, 10 minutes post-injury and 30 minutes post-injury respectively with an overall AUC of 0.83.

## I. Introduction

Hemorrhage accounts for approximately 35% of the mortality from 5 million traumatic injury related deaths annually worldwide, second only to central nervous system injury [1], [2]. In 2015, the national trauma institute estimated that in civilian populations, sever bleeding accounts for greater than 35% of pre-hospital deaths and nearly 40% of deaths within the first 24 hours of injury. Hemorrhage management has also been identified as a priority focus area for the United States military [3] because hemorrhage is the most common cause of preventable death in the battlefield [4]. Hemorrhagic injuries represented 30% of the years of potential life lost before age 65 and accounted for almost 10% of the national expenditure on healthcare. Despite advances in medicine and pre-hospital care, these numbers have not improved significantly.

There are three main categories of bleeds. Lacerations, punctures, and amputations can result in arterial bleeding which is characterized by spurting, or pulsatile, bright red blood, resulting in quick blood loss and death, compared to venous or capillary bleeding [5]. Patients with trauma and severe hemorrhage require timely hemorrhage control combined with resuscitation to replace lost blood volume and mitigate the pathophysiologic consequences of hemorrhagic shock.

The involuntary action for hemorrhage control is to compress the bleeding site by pressure dressings, tourniquets, or by bare manual application of pressure. If the given pressure is high enough, the bleeding will cease, thus termed as compressible hemorrhage and often found in accessible sites, such as the extremities. If there is a hemorrhagic focus that is inaccessible to a tourniquet or pressure dressing, such bleeding is termed as non-compressible hemorrhage. In this case, it is more difficult to break the cycle of bleeding and organ dysfunction [6].

Although known approaches to control non-compressible torso hemorrhage (NCTH) include sponges, pressure devices, and hemostatic agents [7], [8], [9], time-sensitive management of these patients is critical and includes transferring them to surgical facilities within hours of the injury [10]. More recently, high-intensity focused ultrasound (HIFU) has emerged as a promising method for hemorrhage control. HIFU can deliver energy to deep regions of tissue where hemorrhage is occurring, allowing cauterization (burn a part in an attempt to mitigate bleeding) at depth in parenchymal tissues, or in difficult-to-access anatomical regions, while causing no or minimal biological effects in the intervening and surrounding tissues. The high rate of energy delivery to the tissue causes cautery and coagulative necrosis (tissue or cell death due to lack of blood flow) [11].

A major challenge in the application of HIFU is the accurate targeting of therapeutic beam to the location of the bleed. In a porcine study, Martin *et al.* [12] found Doppler ultrasound to be effective for localizing the blood vessel puncture site. Several investigators [13] studying HIFU as an approach for hemorrhage control have since used Doppler ultrasound (US) imaging for guidance as it can provide the direction and velocity of blood flow in arteries. In practice however, the need for an expert sonographer to interpret images in realtime during the delivery of HIFU can significantly limit the utility of this therapy in non-clinical environments. This suggests the need for a method that automatically can interpret the focus of hemorrhage or detect hemorrhage when Doppler US is used by a non-expert sonographer.

Recent advances in machine and deep learning methods for detection and localization tasks have shown promising results in a variety of healthcare applications including hemorrhage detection. However, most of existing hemorrhage detection approaches are either supervised [14], [15], [16], semi-supervised [17] or weakly supervised [18]. Also, most of the existing works are focused on brain intracranial hemorrhage (ICH) detection in head CT scans, and NCTH detection still remains largely unexplored. For supervised learning algorithms, the model learns from a large training set comprised of data and labels from every class of objects the model is subsequently expected to detect. Several supervised learning methods have been proposed before for ICH detection. Some approaches included recurrent attention DenseNet (RADnet) [14], double-branch CNN based hemorrhage feature extraction [16] and CNN with Long Short-Term memory (LSTM) [15], which leveraged both spatial and temporal features from CT slices. However, since the collection of annotations (labels) is often time-consuming and thereby costly as well as in many cases, obtaining a confident ground truth becomes challenging, the usability of supervised learning algorithms become limited.

For semi-supervised and weakly supervised methods, a relatively small set of annotated data are provided for training in addition to large amount of data without annotation. By applying attention mechanisms, Wang *et al.* proposed a semi-supervised multi-task attention-based U-Net for ICH segmentation with small amount of labeled data [17]. Nemcek *et al.* applied weakly supervised learning approach with only class label for ICH localization by detecting local extrema in attention maps from the CNN classification network [18]. However, obtaining hemorrhage data from a bleeding subject has its own challenges as timely intervention becomes a top priority in such cases. Therefore, unsupervised learning algorithms become a feasible choice for automatic hemorrhage detection. There are a few unsupervised methods to detect blood pool in blunt abdominal trauma from Focused Assessment with Sonography in Trauma (FAST) ultrasound. The methods involve image pre-processing, and analysis of local intensity features using K-Means clustering and levelsets [19], [20], [21], [22].

Although these unsupervised methods detected blood pool, these were not specifically designed to detect the location of the hemorrhage captured by color Doppler ultrasound, which showed that jet flow at the site of hemorrhage had a different (red-blue check) pattern probably capturing turbulence compared to the uniform blood flow in the entire artery [23], [24], [25]. This difference in pattern fits the definition of outliers (or anomalies) in the data, thus motivating the application of unsupervised anomaly detection. Anomaly detection methods are generally employed in applications where the goal is to identify instances that deviate from what is considered ‘normal’ and in instances where it is difficult to study the signature of the anomalous class either due to lack of data on account of the statistical rarity of the event needed to produce the data and/or due to the highly heterogeneous nature of what constitutes an anomaly.

In recent years, anomaly detection using medical image data scans typically follow deviation based methods like Auto Encoders (AEs) [26], [27], [28] or generative adversarial networks (GANs) [29], [30], [31] based methods. AE and GAN are two common deep learning network architectures for unsupervised anomaly detection. Both AE and GAN use convolutional kernels, however their applications in the sense of deviation based anomaly detection are fundamentally different, while both are trained using normal/healthy samples. In AE, the encoder typically encodes a sample image into a lower dimensional latent space. The decoder uses this latent space representation to reconstruct the sample such that a deviation between the sample and the reconstruction can be calculated [32]. In GAN, although training a generator and a discriminator in an adversarial setup enables learning a manifold of the normal samples by the generator, an additional iterative process is required to detect anomalies by mapping the unseen anomaly image to the latent space, such that the difference between the input and the reconstructed image highlights the anomalies [29].

Given the challenges of acquiring data for arterial hemorrhages, in this work, we explore the anomaly detection approach using GAN [29], where a deep neural network learns the normal blood flow velocity distribution of an artery from color Doppler ultrasound images of healthy subjects. The trained network then seeks to perfectly reconstruct images that lie within the learned manifold of normal variability. In the event of a hemorrhage, the network will only be able to reconstruct regions of the image that are normal and thereby the residual image can provide pixel-level detection of hemorrhages.

## II. Method

### A. Data

For data collection, a porcine animal model of hemorrhage developed at University of Maryland, School of Medicine (IUCAC protocol number: 0220014, date: 2/21/20) was chosen for this study. N = 11 pigs were imaged using a GE Healthcare Vivid I, linear array probe and color Doppler US. Following normotensive baseline images ( $T_{0}$), hypotension was induced by injuring the femoral artery with an oversized endovascular balloon, creating a region of “active extravasation” (a discharge or escape of blood, from a vessel into the tissues), thus provoking hemorrhage. The vessel was re-imaged immediately ( $T_{1}$), 10 minutes ( $T_{2}$) and 30 minutes post injury ( $T_{3}$) for some animals. All data were stored in GE DICOM format.

### B. COLOR Doppler Us Pre-Processing

Color Doppler images on the ultrasound console are displayed as RGB color overlays on B-mode grayscale images over a small region-of-interest on the anatomy, indicating direction of blood-flow (arteries vs. veins) depending on the flow towards or away from the ultrasound transducer. It is possible to extract the color flow information from the vendor-specific raw DICOM images.

Color flow images were pre-processed using a GE internal pipeline based on [33], [34], and [35]. Derived estimates of blood flow velocity ( $CF_{vel}$) and power ( $CF_{power}$) were computed from the complex color Doppler data embedded within the DICOM tags. The color Doppler data was stored as quadrature components $R_{xx}$ and $R_{xy}$ and power spectrum ( $P$) of the signal. Auto-correlation estimates $R(0,0)$ and $R(0,1)$ at zero and unit lags respectively were computed from the quadrature components and the power spectrum using the equations below:

\begin{align*} P=&10\log (R(0,0)) \tag{1}\\ R(0,1)=&(R_{xx}+iR_{xy})\times R(0,0)\tag{2}\end{align*}

Pixel-wise velocity ( $CF_{vel}$) and power ( $CF_{power}$) estimates were derived from these auto-correlation components and Nyquist’s frequency ( $\mathit {f}_{Nyquist}$) of the signal (obtained from the DICOM tag) as follows:

\begin{equation*} CF_{vel}=\theta (R(0,1))/\pi \times \mathit {f}_{Nyquist}\tag{3}\end{equation*}

where, $\theta $ is the phase angle of the complex $R(0,1)$ autocorrelation estimate, and

\begin{equation*} CF_{power}=R(0,0)\tag{4}\end{equation*}

**FIGURE 1.**
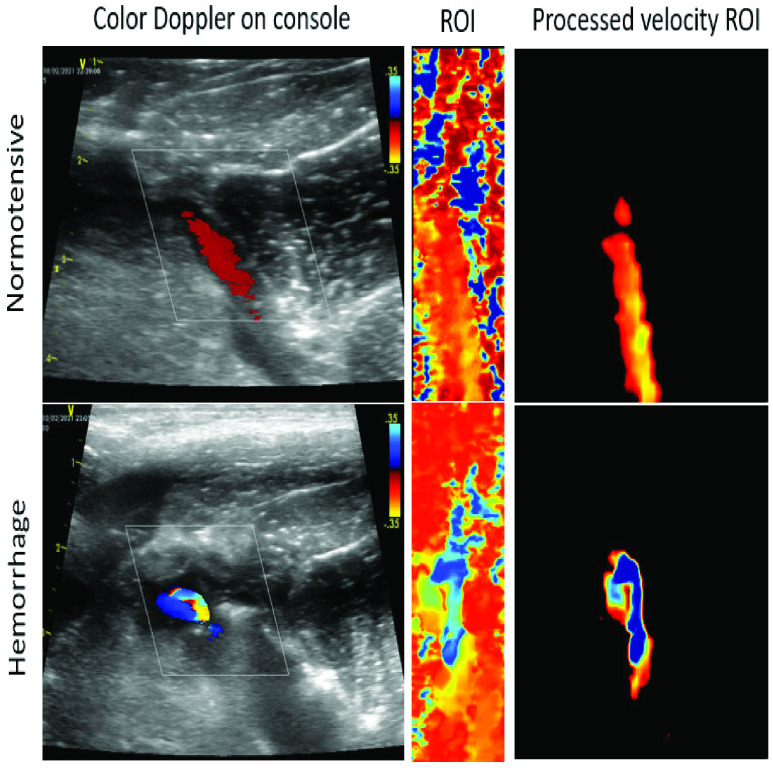
Color doppler ultrasound, extracted ROI on vessel of interest, and pre-processed velocity image as input to the hemorrhage detection network.

The power maps were further thresholded using an Otsu threshold filter and the binary map was used to mask out velocity values in non-arterial and background tissues within the velocity maps. Figure 1 shows the input color Doppler and corresponding pre-processed velocity images. The pre-processed images are shown using a RGB color map for visualization purposes only, the velocity data were actually encoded in 16 bits.

Centering around the largest component in the velocity map, the images were padded with zero values to maintain a 1:1 aspect ratio and then resized to $64\times 64$ pixels. Pixel intensity values were normalized between -1 to 1.

### C. Generative Adversarial Networks

GAN is based on a game-theoretic approach, where the network tries to learn to generate data from a training distribution through a two-player game, where two adversaries or sub-networks, a generator $G(.)$ and a discriminator $D(.)$ are in a constant battle. During the adversarial training process, the generator learns to synthesize realistic images similar to those in the training set while the discriminator learns to distinguish between real and generated images. With a wide adoption of Convolutional Neural Nets (CNN), GAN is a popular deep learning network architecture for generative modeling, where the network learns to estimate the latent space data distribution associated with the training set and then outputs samples generated from that distribution [36]. While GAN has proven to be a powerful technique for data generation, in practice, GAN is unstable to train because two sub-networks are trained from a single backpropagation with combination of generator and discriminator loss. This results in networks that generate unrealistic output images. Deep Convolutional GANs (DCGAN) [37] add architectural constraints to both the generator and discriminator networks in order to reduce training instability and to generate more relevant output images.

### D. Generative Adversarial Networks for Anomaly Detection

In the context of anomaly detection, GANs are only trained with normal images. By training a GAN with normal images, the generator learns a manifold of normal images and knows how to reconstruct normal images, and the discriminator learns to distinguish between real and reconstructed samples. The GAN network for anomaly detection is not trained with any anomalous examples [38]. At testing, when an image with anomaly is presented to this trained generator, the image will still be reconstructed based on the manifold of normal data. However, this will create a residual between the reconstructed and input anomalous query image, which represents the anomaly. Since no supervision is given to the GAN framework regarding how to identify abnormalities, the training process is unsupervised.

In this work, we leverage AnoGAN [29] for hemorrhage detection, which is a GAN network for anomaly detection based on DCGAN architecture. In our experiments, hemorrhage is anomalous blood-flow. The inputs to AnoGAN training is normal blood-flow image and a uniformly sampled, random latent vector. The outputs are reconstructed normal image and a manifold of normal images learned by the generator, while the discriminator evaluates how good the reconstructed image is compared to the input image. At testing, the input to the network is a hemorrhage image and the output is a reconstructed hemorrhage image based on the learned manifold by the generator, plus the residual image that represents the anomaly i.e., the difference between input hemorrhage image and the reconstructed image.

**FIGURE 2.**
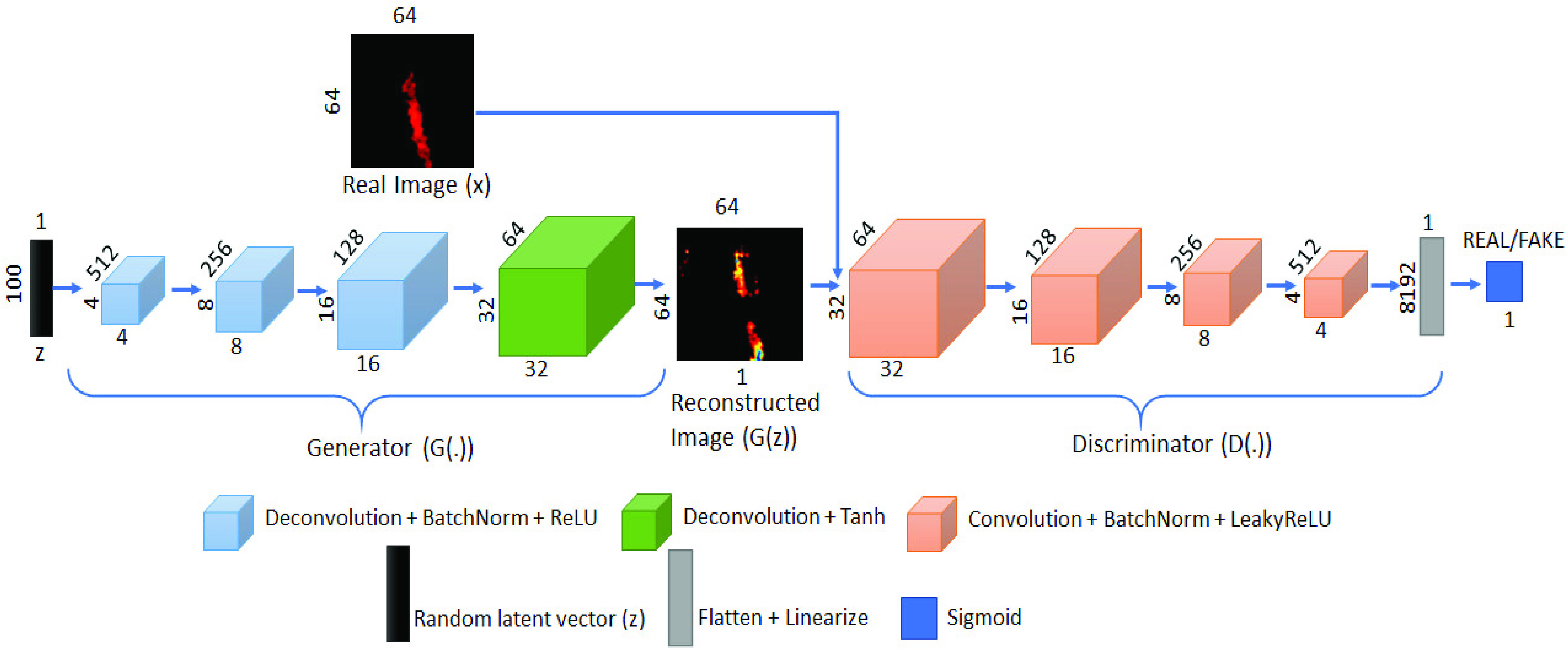
Deep convolutional generative adversarial (DCGAN) network for color doppler ultrasound based hemorrhage detection.

Figure 2 provides a schematic of the deep learning network architecture, which is similar to our previous work [39], and is composed of two sub-networks: generator and discriminator. The generator in this architecture consists of four sequential deconvolution blocks that upsample and map low dimensional latent vector $z$ to high dimensional images x. Each of first three blocks contains fractionally strided deconvolution, batch normalization and ReLU (Rectified Linear Units) activations, and the last block is composed of fractionally strided deconvolution and Tanh (hyperbolic tangent) activation function. The generator takes the 1D latent input noise vector $z$ with size of 100, and it performs the non-linear mapping via $G(z)$ and learns the distribution $p_{G}$ over the real image x onto a 2D manifold with the dimensions of the real image. The discriminator $D(.)$ consists of four standard convolution blocks with LeakyReLU activations, which downsample the generated images into a flattened layer and then linearize into a scalar output. Subsequently, the sigmoid activation function classifies the generated $G(z)$ as a ‘real’ or ‘fake’ image.

In the training, the discriminator $D$ is trained to maximize the probability of classifying the real training images as ‘real’ and generated images as ‘fake’ label. The generator $G$ is simultaneously trained to fool discriminator by minimizing $V(G)$ = $log(1-D(G(z)))$. During the adversarial training process, the generator improves in generating realistic images and the discriminator progresses in correctly identifying real and generated images [29]. The training of the network is achieved by maximizing the function $V(G,D)$ as a min-max game, and the objective function can be written as,

\begin{align*} \mathop {\mathrm {min}}_{G} {\mathop {\mathrm {max}}_{D} V(G,D) \!=\! E_{\mathrm {x}}{\mathrm {\big [log} \big (D(\mathrm {x})\big)\big] \!+\! E_{z}{\mathrm {\big [log} \big (1\!-\!D\big (G(z)\big)\big)\big]}}}\!\! \\\tag{5}\end{align*}

After the generator has completed training, an iterative method is used for anomalies detection, as described in algorithm 1. To map new images x to latent space vector $z$, a randomly sampled latent vector $z$ from the latent space $Z$ is mapped to $G(z)$ via the trained generator, which outputs a generated image. The position of $z$ is updated to find the most similar image $G(z_{\theta })$, and this update is performed over $\theta =1,2, {\dots }\varphi $ backpropagation steps. The loss function for backpropagation in mapping the new image to the latent space is defined by 2 components, the residual loss $\mathcal {L}_{\mathcal {R}}$ and the discrimination loss $\mathcal {L}_{\mathcal {D}}$ for $z_\theta $.

\begin{align*} {\mathcal {L}}_{\mathcal {R}}(z_{\theta })=&\sum {|\mathrm {x}-G(z_{\theta })|} \tag{6}\\ {\mathcal {L}}_{\mathcal {D}}(z_{\theta })=&\sigma (D(G(z_{\theta })),\alpha)\tag{7}\end{align*}

Algorithm 1 **Iterative Anomalies Detection**
**Require:** Input query image x
**Require:** Total iteration number $\varphi $
**Require:** Trained generator network $G(z)$
**Require:** Trained discriminator network $D(\mathrm {x})$
1: randomly sample ${z}$ from latent space distribution ${Z}$
2: **for** $\theta = 1$, $\theta {+}{+}$, while $\theta < = \varphi $ **do**
3: generated image: $\mathrm {x}_\theta \gets ~G(z)$
4: residual loss: $\mathcal {L}_{\mathcal {R}_\theta } \gets ~\mathcal {L}_{\mathcal {R}}(\mathrm {x}, \mathrm {x}_\theta)$
5: discrimination loss: $\mathcal {L}_{\mathcal {D}_\theta } \gets ~\mathcal {L}_{\mathcal {D}}(\mathrm {x}, \mathrm {x}_\theta)$
6: total loss: $\mathcal {L}_\theta \gets \,\,(1 - \gamma)\mathcal {L}_{\mathcal {R}_\theta } + \gamma \mathcal {L}_{\mathcal {D}_\theta }$
7: update ${z}$ based on total loss $\mathcal {L}_\theta $
8: **end for**
9: anomaly score: $A \gets \,\,(1 - \gamma)\mathcal {L}_{\mathcal {R}_\varphi } + \gamma \mathcal {L}_{\mathcal {D}_\varphi }$
10: residual: $\mathcal {R} \gets \,\,|\mathrm {x}_{\varphi } - \mathrm {x}|$
11: **return** ${\mathcal {R}}, A$

Residual loss measures the visual dissimilarity between the new image x and the generated $G_{z_\theta }$, while the discrimination loss is defined as the sigmoid cross entropy ( $\sigma $) of generating real images with logits $D(G(z_{\theta }))$ and targets $\alpha =1$. Finally, the total loss to update the latent vector position of $z_\theta $ to $z_\varphi $ is defined by:

\begin{equation*} \mathcal {L}(z_{\theta })=(1-\gamma){\mathcal {L}}_{\mathcal {R}}(z_{\theta })+\,\,\gamma {\mathcal {L}}_{\mathcal {D}}(z_{\theta })\tag{8}\end{equation*}

where $\gamma $ is an empirically determined parameter between 0 and 1. Only the coefficients of the latent vector $z_\theta $ are updated at this stage, while the trained generator and discriminator coefficients remain fixed.

Anomaly score, $A(\mathrm {x)\,\,=\,\,}\mathcal {L}(z_\varphi)$, is computed as the loss described in (8) at the final $\varphi ^{th}$ backpropagation step. If the input query image x has anomalies i.e., different from the distribution of the trained images, a large anomaly score will be generated between 0 and 1.

## III. Experiments and Results

**FIGURE 3.**
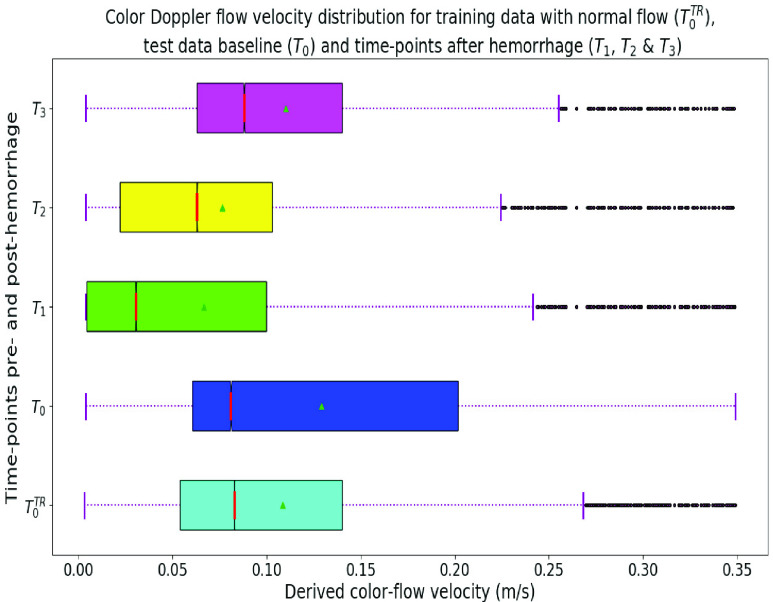
Distribution of color-flow velocities for normal baseline of both training and test data, and hemorrhage time-points of test data. ’Red’ bars represent the medians and ‘green’ triangles show the means of distributions.

A total of 11 pigs were used for our experiments. The normotensive images (n = 3402) from 8 pigs were used to train the AnoGAN. This is an extended dataset compared to 5 pigs used in training and evaluation in our previous work [39]. The network was trained for 25 epochs using Adam optimizer with a learning rate of 0.0002. For testing, images from 3 pigs were used that were not used in training. The baseline normotensive images (n = 712) at ${T_{0}}$ were available for all 3 pigs. Hemorrhage images (query) at ${T_{1}}$ (n = 454) and ${T_{2}}$ (n = 305) were available for 1 pig, and ${T_{3}}$ (n = 370) hemorrhage images were available for another pig. It is to be noted that timepoint ${T_{3}}$ was not evaluated in [39]. Figure 3 shows the flow velocity distribution derived from non-zero pixels in the velocity component of color Doppler images for training data with normal flow ( $T_{0}^{TR}$), and time-points of test data from baseline (normal flow) and after hemorrhage i.e. ${T_{0}}$ to ${T_{3}}$.

During testing, 2000 backpropagation steps were used to map each input image to the latent space. $\gamma $, the weighting factor of residual and discriminator losses was empirically set to 0.1. A set of residual images were generated defined as the difference of the input query image and reconstructed query image based on the latent normotensive distribution. The residual images were thresholded at a value determined from the histogram of all residual images. This generated residual binary images (white pixels for hemorrhage location with black background). The residual images were also thresholded at different values to generate ROC curves that measures the performance of anomaly detection for hemorrhages when compared to baseline without hemorrhage.

**FIGURE 4.**
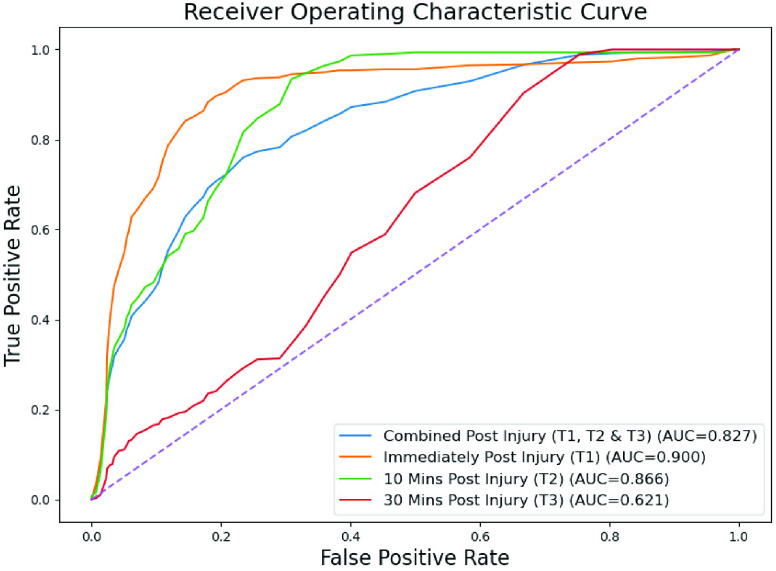
Receiver operating characteristic (ROC) curve for hemorrhage detection at ${T_{1}}, {T_{2}}, {T_{3}}$ and combination of all.

Performance statistics of the hemorrhage detection are summarized in Table 1, and the corresponding ROC curves are shown in Figure 4. The sensitivity of our method in detecting hemorrhages at time-points ${T_{1}}, {T_{2}}, {T_{3}}$ were 93.6%, 84.5% and 31% respectively, while the specificity of the method was 74.4%. The AUC for time-points ${T_{1}}, {T_{2}}, {T_{3}}$ were 0.9, 0.87 and 0.61 respectively, with a mean AUC of 0.83.

**TABLE 1:** Performance Statistics [Sensitivity, Specificity and Area Under Curve (AUC)] for Hemorrhage Time-Points ${T_{1}}$, ${T_{2}}$, ${T_{3}}$

Imaging Time	Sensitivity	Specificity	AUC
Immediately Post Injury ( ${T_{1}}$)	93.6%	74.4%	0.90
10 Minutes Post Injury ( ${T_{2}}$)	84.5%	74.4%	0.87
30 Minutes Post Injury ( ${T_{3}}$)	31.1%	74.4%	0.61

**FIGURE 5.**
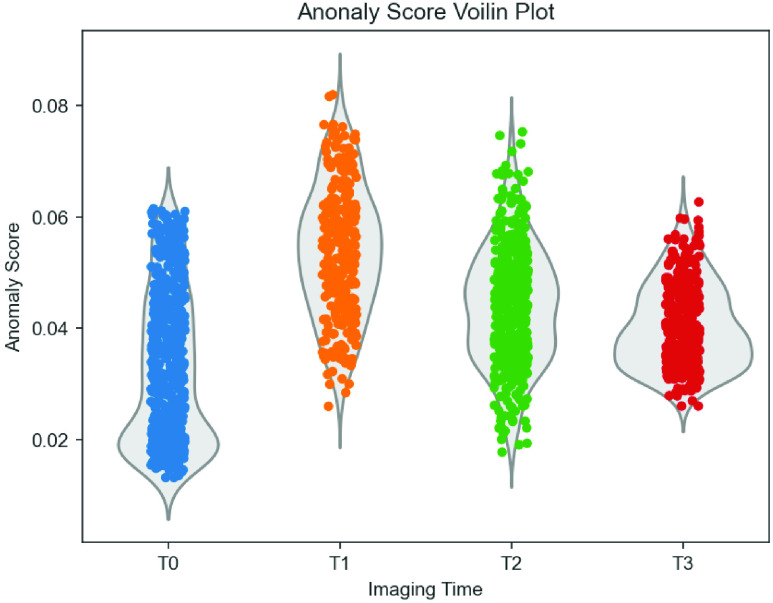
Anomaly score distribution for baseline ${T_{0}}$, and hemorrhages at ${T_{1}}, {T_{2}}$ and ${T_{3}}$ respectively. The distribution of hemorrhage anomaly scores were statistically significantly different from normal at $p < 0.0001$.

The anomaly score distributions for different time-points ${T_{0}}, {T_{1}}, {T_{2}}, {T_{3}}$ are shown in Figure 5. The mean anomaly score defined in (8) between the ${T_{0}}, {T_{1}}, {T_{2}}$ and ${T_{3}}$ images were 0.035± 0.017, 0.044± 0.011, 0.054± 0.011 and 0.040± 0.007 respectively. The anomaly score distribution of all hemorrhage time-points were significantly different from the baseline( $T_{0}$) at 95% CI, Bonferroni-corrected for multiple comparisons at $p < 0.0001$.

**FIGURE 6.**
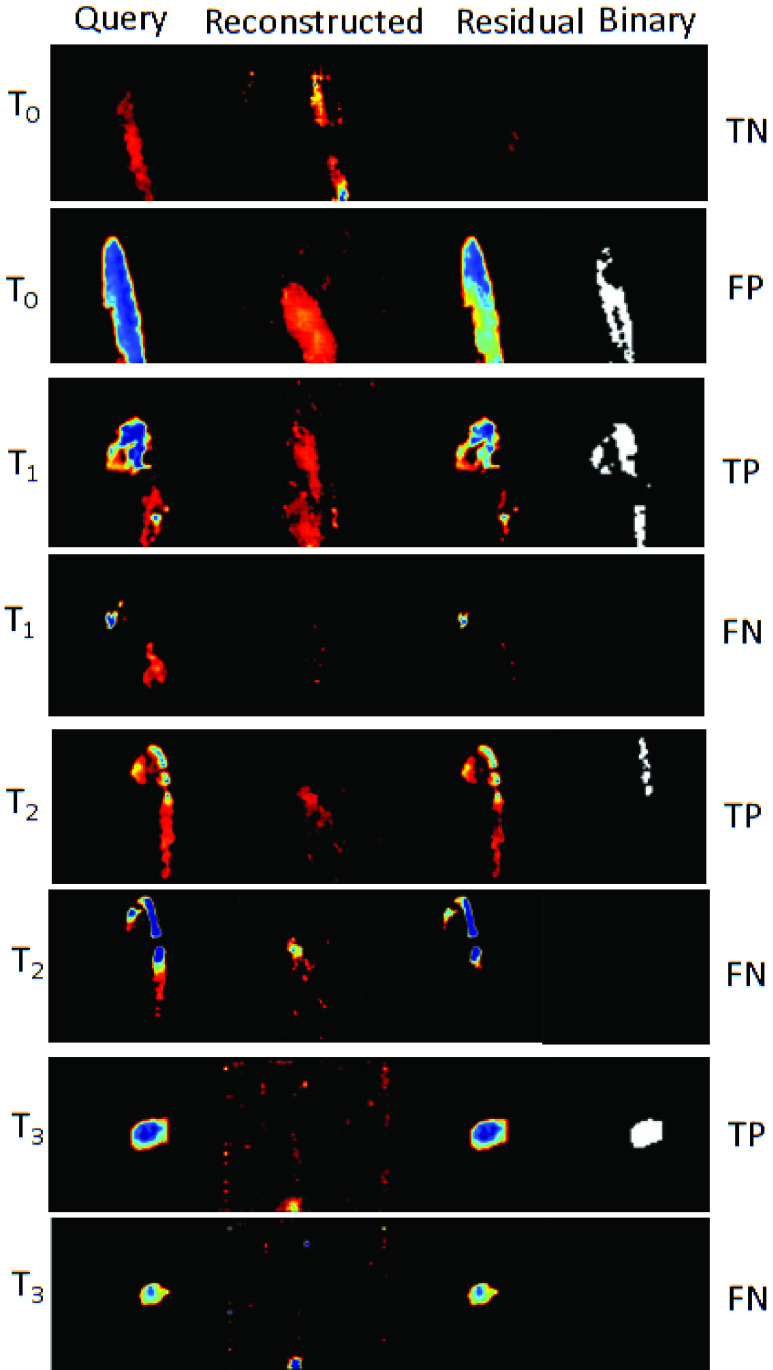
Reconstructed and residuals of the query images. First column is the query image, second column is the reconstructed image, third column is the residual image, and the last column is the binarized residual image. ${T_{0}}, {T_{1}}, {T_{2}}, {T_{3}}$ are the normotensive, immediately after hemorrhage, 10 minutes and 30 minutes post hemorrhagic conditions. TP, TN, FP and FN are the true-positive, true-negative, false-positive and false-negative respectively.

Figure 6 shows the query images, reconstructed query images, the residual images, and the binarized residual images. Ideally for T0 normotensive images, the residual binary should be a blank image (true negative-TN) and for a query hemorrhage image the residual binary should show the hemorrhage focus in white pixels (true positive-TP).

## IV. Discussion

To the best of our knowledge, this is the first study to attempt detecting hemorrhagic focus automatically in ultrasound color Doppler images for immediate attention and possible cauterization of the artery to prevent blood loss. Previous studies [19], [20] have attempted automatic detection of blood pool from ultrasound images for emergency diagnosis in the event of blunt traumatic injury using a machine learning framework. There have been several studies [40], [41] attempting to detect and localize cranial hemorrhages using deep learning from CT images after traumatic injury. Moreover, previous studies using deep learning or machine learning-based method for hemorrhage detection used supervised techniques, while our method involves unsupervised identification of hemorrhage.

The results in Figure 4 demonstrate the generative capability of the AnoGAN based anomaly detection network to detect hemorrhages of the femoral artery for both immediately after injury ${T_{1}}$ and 10 minutes post injury ${T_{2}}$. Figure 5 further corroborates appropriateness of the proposed anomaly scoring approach for hemorrhage indications. A reduced sensitivity of the method was however noticed for ${T_{3}}$ i.e. 30 minutes post-injury in Figure 4. This can be partly explained by Figure 3 where, despite outliers in flow velocity distributions at ${T_{3}}$, the overall distribution matches the range of training data normal flow velocities ( $T_{0}^{TR}$). This explains the failure of the hemorrhage detection method at ${T_{3}}$ that relies on the concept of anomaly, i.e. what deviated from normal. Similarly, the low specificity of the method can explained by the distribution difference of normal blood flow velocities between training data ( $T_{0}^{TR}$) and test data ( ${T_{0}}$) as these were from different animals.

We believe our method specifically captured the turbulence of blood flow at near time-points post-injury ( ${T_{1}}$ and ${T_{2}}$) as the oxygenated blood spurted out through the site of arterial injury with increased systolic and diastolic velocities causing flow turbulence. It can be explained as the converging blood at injury causing contradictive angles between the flow and Doppler beam that cause the non-uniform pattern at site of arterial injury [25]. The red-blue check patterns observed in Figure 6 further corroborate the findings of [23], [24] related to blood flow turbulence at arterial injury site. At time-point ${T_{3}}$, we noticed a reduced turbulence (lacking representative red-blue check pattern) at arterial injury site.

Several clinical factors may have had effects on the arterial behavior and thus flow at different timepoints. Immediate effects of vasospasm (narrowing of brain blood vessels to block blood flow) as a wire and balloon traveling through the artery was used to induce bleeding may have been present at ${T_{1}}$ and ${T_{2}}$. Similarly, driven by sympathetic response, a physiological compensation by increasing the heart rate and mean arterial pressure may have happened immediately at ${T_{1}}$ and ${T_{2}}$ and have offseted after minutes at ${T_{3}}$. The blood volume may have decreased rapidly due to bleeding immediately after the injury but there is a compensation to replace the lost volume as the interstitial and intercellular volume are moved into the intravascular space, which happens after few minutes of bleeding. A similar study performed by Madurska *et al.*, [42] to study the effects of hemorrhage on hemodynamic indices showed that the greatest fall in mean hemodynamic indices were noted in the first 15 min of hemorrhage, which is approximately ${T_{2}}$ of our study. As these clinical parameters balance-off over time post-hemorrhage, we may see a new normal in hemodynamic indices at ${T_{3}}$, which is still not the baseline ${T_{0}}$. These findings further suggest the need for an automatic hemorrhage detection method that can capture turbulence in early stages of hemorrhage to prevent the subject from a hemorrhagic shock or death.

The interpretation of the AnoGAN residual for identifying hemorrhage focus in each color Doppler US frame is slightly different in our work compared to methods in [38] and [29]. While anomaly constituted regions within normal areas of images in previous studies, in our study each image is either hemorrhage or normal. As our method has a lower specificity (74%) compared to sensitivity of hemorrhage detection, the interpretation of hemorrhage or no-hemorrhage can be made over a sequence of images or color Doppler movie frames. If the appearance of residuals over the movie frames is high i.e., almost every frame has residual regions, the interpretation would be hemorrhage or no-hemorrhage otherwise. This would enable automated interpretation of hemorrhage in color Doppler US.

A major disadvantage of the AnoGAN network used in our method is that it requires training of an encoder to map the query image into latent space at the time of testing, which is a computationally intensive optimization process that does not allow quick adoption of the method for real-time interventions. As part of our future work we plan to leverage the f-AnoGAN [30] network architecture for faster inference of color flow images. However, in future, if a few labeled images of hemorrhage are available, we may adopt a semi-supervised learning approach where residual attention modules or attention maps may be used for detection of hemorrhage similar to [17] and [18].

In our work, we used only images of femoral artery as part of our feasibility study, which is usually easy to image under ultrasound. As a next step, we plan to train the network with images from complex anatomy such as abdominal vessels, i.e. the network will be trained with the normal blood flow of several vessels from different locations to learn the flow distribution. As our method has shown promise in capturing turbulent blood flow, with the query hemorrhage image as input, we believe our network with f-AnoGAN [30] that uses Wasserstein GAN [43], [44] as the underlying architecture (measuring Wasserstein distance between the generated and the real images) will still be able to differentiate turbulence from normal blood flow given the large distribution of normal flow velocities across different arteries. As an alternative, we can also use a multi-generator GAN [45] approach where, disconnected manifolds are learned instead of one smooth manifold while minimizing the mutual information across different generators as the generator loss.

The results presented in our work are important to be considered in clinical context. Patients with life threatening bleeding progress through stages of physiological compensation, and if left untreated leads to eventual decompensation (functional deterioration of a structure or system) and death. The greatest opportunity for successful intervention lies in the early stages of shock, often when its initial presentation is occult. If an insult can be identified and treated prior to decompensation, then patients are much more likely to survive [42], [46]. Unfortunately, clinical signs are most obvious in the terminal stages of shock. The strength of the presented algorithm lies in its accuracy of early detection, providing clinicians with a tool to exploit a window of early detection. However, a successful translation of this tool in trauma care not only requires extensive clinical validation through trials, the accurate placement of the ultrasound probe closer to the site of injury, particularly for internal hemorrhages, which probably requires another intermediate method to localize approximate site of injury based on pulse wave reflections [47]; the availability of laptop-based ultrasounds equipped with graphics processing unit with the hemorrhage detection algorithm embedded for fast localization of hemorrhage are some of the aspects that need further development.

## V. Conclusion

We investigated an unsupervised GAN-based anomaly detection network to detect blood flow anomalies associated with hemorrhage on ultrasound color Doppler images. The network was trained on femoral artery images collected from a limited set of pigs in normotensive conditions, and it accurately reconstructed normal arterial blood flow and provided low anomaly or residual scores on an independent set of normotensive data. When presented with color Doppler images at the site of hemorrhage, the network provided statistically significant high anomaly scores for all the time-points compared to the baseline normal. The method shows promise in localizing anomalies at pixel level and in presenting the focus with high sensitivity before the subject goes to hemorrhagic shock. These results suggest that the presented approach may play a key role in enabling HIFU or other image-informed intervention for hemorrhage management at the point of care, in the absence of an expert sonographer.
